# Deep rTMS Mitigates Behavioral and Neuropathologic Anomalies in Cuprizone-Exposed Mice Through Reducing Microglial Proinflammatory Cytokines

**DOI:** 10.3389/fnint.2020.556839

**Published:** 2020-11-05

**Authors:** Liu Yang, Yawen Su, Fannv Guo, Handi Zhang, Yinglin Zhao, Qinjun Huang, Haiyun Xu

**Affiliations:** ^1^The Mental Health Center, Shantou University Medical College, Shantou, China; ^2^The School of Psychiatry, Wenzhou Medical University, Wenzhou, China

**Keywords:** cuprizone, microglia, oligodendrocyte, rTMS, inflammatory cytokines

## Abstract

In comparison to conventional repetitive transcranial magnetic stimulation (rTMS), theta burst stimulation is stronger and more effective as a brain stimulation approach within short periods. Although this deep rTMS technique is being applied in treating neuropsychiatric disorders, few animal studies have attempted to clarify the neurobiological mechanisms underlying its beneficial effects. This animal study examined the effects of deep rTMS on the cuprizone-induced neuropathologic and behavioral anomalies and explored the underlying mechanism. Adolescent male C57BL/6 mice were fed a rodent chow without or with cuprizone (CPZ; 0.2% w/w) for 5 weeks. Another two groups of mice were subjected to deep rTMS or sham rTMS once a day during weeks 2–5 of the CPZ-feeding period. The behaviors of all mice were assessed after the withdrawal of CPZ before neuropathological and immunological analyses. Compared to the CNT group, mice in CPZ and CPZ + Sham groups showed deficits in social recognition and spatial working memory as well as anxiety-like behavior, in addition to myelin breakdown and OL loss in the corpus callosum (CC), caudate putamen, cerebral cortex, and hippocampus of the brain. Deep rTMS effectively reduced behavioral anomalies and blocked myelin breakdown and OL loss in CPZ-fed mice. Besides, it also dampened microglia activation at lesion sites and rectified cytokines levels (IL-1β, IL-6, and IL-10) in CPZ-affected regions. The most significant effect was seen in the cerebral cortex where alleviated neuropathology co-existed with less microglia activation and higher IL-10 level. These data provided experimental evidence for the beneficial effects of deep rTMS in CPZ-fed mice and revealed a neurobiological mechanism of the modality.

## Introduction

Transcranial magnetic stimulation (TMS) is a non-invasive neuromodulation technique. *Via* electromagnetic induction, transient and localized electrical fields are generated in the brain cortex, which in turn affect functions of local neurons such as depolarization and firing (Hallett, 2000). Repetitive TMS (rTMS), as the term implies, delivers multiple TMS pulses at frequencies between 0.5 and 20 Hz over a chosen brain area (Pascual-Leone et al., 1999). Moreover, the theta-burst stimulation (TBS, a newer modality of rTMS) has been developed and applied in humans. TBS delivers relatively greater and more stimulation to the brain in a shorter period (Huang et al., 2005; Chung et al., 2015). This deep rTMS was shown to change cortical excitability that may last longer than with traditional TMS protocols (Ishikawa et al., 2007; Huang et al., 2009). As reported by Levkovitz and colleagues, TBS led to higher response and remission rates in 212 major depressive depression outpatients than in the sham group (Levkovitz et al., 2015). Recent evidence from human studies supports a therapeutic role of TBS in some neuropsychiatric disorders, which implicates certain brain regions important to cognitive functions (Spronk et al., 2011).

In contrast to its growing clinical applications in patients with neuropsychiatric disorders, few animal studies have been done on deep rTMS applications. Of the extant publications, Zhang et al. (2014) reported the beneficial effects of deep rTMS on hippocampal neurogenesis and the development of adult newborn neurons, in addition to its anti-depression effects seen in the learned helplessness mouse model and a mouse model for Rett syndrome. In a more recent study, administration of deep rTMS (gamma Hz) for 6 weeks alleviated schizophrenia-like behaviors in cuprizone (CPZ) exposed mice while promoted the remyelination process and up-regulated the expression of neuregulin-1 and its receptor ErbB4 in the prefrontal cortex (PFC) of the demyelinated mice (Sun et al., 2018).

CPZ is a chemical chelator that is toxic to mitochondria by inhibiting the activity of complexes I-IV in the mitochondrial respiratory chain (Pasquini et al., 2007; Millet et al., 2009; Acs et al., 2013). Feeding C57BL/6 mice with a 0.2% CPZ-supplemented diet for 4–6 weeks was shown to induce acute demyelinating lesions followed by spontaneous remyelination in the brains of subjects (Hiremath et al., 1998; Matsushima and Morell, 2001). In addition to demyelination and loss of mature oligodendrocytes (OLs, the myelin-forming cells in the brain), CPZ-exposed mice also showed astrocyte proliferation and microglia activation in the lesion sites of the brain (Stidworthy et al., 2003; Remington et al., 2007; Zhang L. et al., 2018). These neuropathological features constitute the constructive validity of CPZ-exposed mice being used as an animal model of multiple sclerosis (MS), a major demyelinating disease of the central nervous system.

In 2009, we first proposed to use the CPZ-exposed mice as a novel animal model in biological psychiatry research of schizophrenia based on multiple lines of clinical and experimental evidence (Xu et al., 2009). Not surprisingly, we found that CPZ-exposed mice displayed more climbing behavior and lower prepulse inhibition at weeks 2 and 3 of the CPZ-exposure period along with higher dopamine but lower norepinephrine levels in the PFC. CPZ-exposed mice presented less social interaction in the presence of evident brain demyelination, myelin breakdown, and OL loss at weeks 4 and 6. CPZ-exposed mice spent more time on the open arms of an elevated plus-maze and exhibited spatial working memory impairment at all time points (Xu et al., 2009). Since then, the CPZ-exposed mice have been employed as an animal model of schizophrenia to explore the pathophysiology of this brain disorder and investigate the neurobiological mechanisms underlying the therapeutic effects of antipsychotic treatment (Xu et al., 2010, 2019; Herring and Konradi, 2011; Liu et al., 2017; Sun et al., 2018; Hayakawa et al., 2019).

Unlike the other animal models of schizophrenia, the brain neuropathology of CPZ-exposed mice involves mitochondrial dysfunction, neuroinflammation, and white matter damage, three essential players in the pathogenesis of schizophrenia (Najjar and Pearlman, 2015; Xu et al., 2016; De Picker et al., 2017; Buckley, 2019). With this CPZ mouse model, our recent studies have explored the anti-inflammation and anti-oxidative stress effects of quetiapine, an atypical antipsychotic (Shao et al., 2015; Xuan et al., 2015). On the other hand, the antioxidant *N*-acetylcysteine protected mature OLs against the toxic effects of CPZ through its antioxidant and anti-inflammatory actions (Zhang L. et al., 2018). In line with this animal study, clinical studies reported that atypical antipsychotics clozapine and risperidone decreased serum levels of the cytokines IL-2, IL-6, and TNF-α (Lu et al., 2004) and showed the antipsychotic effect of minocycline, a potent inhibitor of microglial activation in patients with schizophrenia (Miyaoka et al., 2007, 2008). Similarly, Celecoxib, a non-steroidal anti-inflammatory drug, appears to be an efficacious and safe treatment in improving psychotic symptoms, particularly in first-episode schizophrenia (Zheng et al., 2017). These animal models and clinical studies suggest the presence of additional therapeutic actions of the extant antipsychotics and encourage further efforts to explore new antipsychotic medications aside from the existing antipsychotics.

This study aimed to examine the effects of deep rTMS on the neuropathologic changes and behavioral anomalies in CPZ-exposed mice and explore the underlying neurobiological mechanism. In addition to assessing the behaviors relevant to anxiety, spatial working memory, and social interaction symptoms seen in patients with schizophrenia, we emphasized CPZ-induced neuropathological changes in the brain. Specifically, we were interested in the role of neuroinflammation in the pathogenesis of CPZ-induced demyelination and OL loss and wondered if deep rTMS works on cells and molecules relevant to neuroinflammation in the brain. The brain regions of the corpus callosum (CC), caudate putamen, frontal cortex (FC), and hippocampus were closely looked at given that these regions are sensitive to CPZ-intoxication (Yang et al., 2009) and compromised in schizophrenia patients (van Erp et al., 2016; Grazioplene et al., 2018). Administration of the deep rTMS for 4 weeks effectively mitigated the behavioral anomalies, reduced myelin breakdown and OL loss in CPZ-fed mice, inhibited microglia activation in the lesion sites and rectified levels of IL-1β, IL-6, and IL-10 in the brain regions mentioned above. These results support the application of deep rTMS in clinical practice and help to understand the beneficial or therapeutic effects of this physical modality in treating schizophrenia patients.

## Materials and Methods

### Animals

Five-week-old male C57BL/6 mice were purchased from the Guangdong Medical Laboratory Animal Center (Guangzhou, China). The mice were housed in groups in polypropylene cages and acclimatized for 1 week before the experiment. The vivarium was kept at 50 ± 5% humidity, 22 ± 1°C, 12 h light-dark cycle. In the vivarium, mice had free access to food and water during the experimental period. All experimental procedures applied to this study were approved by the Local Ethical Committee for Animal Experiments, Shantou University Medical College.

### Experimental Design

After acclimatization for 7 days, the mice were randomly assigned into one of the following four groups (*n* = 14/group): control group (CNT), in which mice received a standard rodent chow and tap water during the experimental period; CPZ group, in which mice ate a CPZ-containing (0.2% w/w) chow for 5 weeks as described previously (Xu et al., 2009); CPZ + rTMS group, in which mice consumed the same CPZ-containing chow as those in CPZ group, plus deep rTMS (described later) during weeks 2–5; CPZ + Sham group, in which mice consumed the same CPZ-containing chow as those in CPZ group, plus sham stimulation, i.e., the cage was set up with the same magnetic equipment at working but no magnetic field was outputted. After consumed CPZ-containing chow for 5 weeks, the mice turned to the normal rodent chow (without CPZ) for 3 days during which period they were subjected to the behavioral tests of open-field, social recognition, and Y-maze in the order, one test per day. One day after the last test, mice were euthanized with sodium pentobarbital (50 mg/kg, i.p.). Seven animals in each group were used for enzyme-linked immunosorbent assay (ELISA) analysis (described later). The remaining seven mice in each group were used for immunohistochemical and immuno-fluorescent staining.

### Deep rTMS Procedure

By referring to previous studies (Zhang et al., 2014; Sun et al., 2018), active deep rTMS or sham rTMS was given to mice for successive 4 weeks during which period mice were fed a CPZ-containing diet. The magnetic equipment (Antis Magnetic Stimulation System, Model: Antis-w-180; Tianjin Antis Medical Device Company Limited, Tianjin, China) with two 360 mm-diameter coils was connected to a magnetic field generator which outputted the so-called intermittent gamma burst stimulation (iGBS) at 30–40 Hz. The iGBS consisted of magnetic fields that changed every 4 min (between linear and uniform gradients) and the rhythm gradually increased (30, 32.25, 34.5, 37, or 40 Hz) during the linear gradient. The 2-min linear gradient was composed of several magnetic pulses, each of which lasted for 2 s, followed by a resting interval for 8 s. Each of the 2-s magnetic stimulations was composed of six pulses with 150 μs width at 1,000 Hz frequency.

### Behavioral Tests

One day after the 5 weeks of CPZ-exposure, all CPZ-exposed mice and normal controls were subjected to the open-field test, social interaction test, and Y-maze test, once a day in the order (Xu et al., 2009). For the open-field test, the mouse was placed in the central zone of an open-field box (25 × 25 cm) and allowed to move freely for 8 min, of which the first 3 min were defined to be the adaptation period and the data from this period were not included for analysis. The travel path of the animal was recorded by a video camera above the arena. The distances traveled on the whole arena and the central zone (12.5 × 12.5 cm), as well as the time spent at the central zone, were analyzed by a video tracking system (DigBehav System, Yishu Co. Ltd., Shanghai, China). The open field was cleaned with 70% alcohol after each trial.

The social interaction test was performed in the same open-field box. The test consisted of two sessions, each lasted for 150 s. During the first session, an empty wire mesh cage (7 × 7 cm) was placed at one corner of the field while a tested mouse moved there freely. During the second session, the conditions were identical to the first session except that an unfamiliar conspecific had been introduced into the cage. Between the two test sessions, the tested mouse was removed from the arena and placed back into his home cage for approximately 60 s. The unfamiliar conspecific was used one time only. The time spent by the tested mouse at the “interaction zone” (a 5-cm-wide corridor surrounding the cage) under the two conditions of an empty cage (E) and the cage with an unfamiliar conspecific (C) was recorded and analyzed after the test.

In the Y-maze test, each mouse was placed at the junction area of the three equal arms (30 × 8 × 15 cm) of a Y-maze and allowed to move freely through the maze for an 8-min test period. The series of entries and the total number of visiting arms were recorded. An actual entry was defined as all four paws of a mouse entered into an arm. A correct alternation was counted if visits into all three arms are in consecutive order. The theoretical maximal number of correct alternations was the total number of arm entries minus 2. The spontaneous alternation was calculated as the number of correct alternations divided by the theoretical maximal number of correct alternations and expressed as a percentage (%).

### Immunohistochemical and Immunofluorescent Staining

Half (*n* = 7) the mice in each group were deeply anesthetized with sodium phenobarbital (50 mg/kg) and perfused intracardially with sterilized saline, followed by 4% paraformaldehyde in phosphate buffer (0.01 M, pH = 7.4). The mouse brain was removed from the skull and post-fixed in the same fixative at 4°C, overnight, and then cryoprotected in 15, 25 and 30% sucrose solutions for 1 day per concentration. Serial coronal sections (25 μm) were cut using a cryostat microtome (Leica CM1850) and collected in six-well plastic plates.

For immunohistochemical staining, the floating brain sections were washed with 0.01 M PBS for 10 min × 3 and then steeped in PBS with 3.0% hydrogen peroxide at room temperature for 30 min. After rinsed in PBS, the sections were incubated with a blocking solution (5% goat serum, 0.3% Triton X-100 in PBS) for 30 min at room temperature. The primary antibody to myelin basic protein (MBP; 1:200; Abcam, Cambridge, UK) or pi form of glutathione-S-transferase (GST-π; 1:200; Boster Biotechnology, Wuhan, China) was added into the blocking solution and incubated at 4°C overnight. After rinsed in PBS, sections were then incubated with the secondary antibody conjugated with horseradish peroxidase (Zhongshan Gold Bridge Biology Company, Beijing, China) at 37°C for 30 min. Then, the sections were visualized by adding a diaminobenzidine solution (Zhongshan Gold Bridge Biology Company). After rinsed in PBS, the sections were pasted onto the glass slides, dehydrated through gradient ethyl alcohol, and cleared by xylene. After cover-slipped with neutral balsam and dried, the immunohistochemical staining was observed and recorded with a Zeiss microscope (Zeiss Instruments Inc., Oberkochen, Germany). The quantitative analysis of the recorded images was done using the Image-Pro-Plus 6.0 software (Media Cybernetics, Rockville, MD, USA).

For immunofluorescent staining, the free-floating sections were washed with PBST (0.5% Tween 20 in PBS) for 5 min × 3. Then the sections were incubated with the blocking solution (5% BSA in PBST) on a shaker for 60 min at room temperature. Subsequently, the primary rabbit polyclonal anti-IBA-1 (1:1,000; Wako, Osaka, Japan) was added into the blocking solution and the incubation continued overnight at 4°C. After rinsed in PBST for 5 min × 3, the sections were incubated with Cy3-conjugated goat anti-rabbit antibody (Beyotime Biotechnology, Shanghai, China) at room temperature for 60 min. After rinsed in PBST, the sections were pasted and mounted by the Fluor mount (Abcam, Cambridge, UK). The immunofluorescence was observed under a fluorescence microscope (Zeiss Instruments Inc., Germany) and images were recorded with the system. The slides were preserved in a refrigerator at 4°C and shielded from light.

### Image Analysis

For quantitative analysis of the immunohistochemical and immunofluorescent staining, all recorded images were read out and analyzed by the Image-Pro Plus 6.0 software (Media Cybernetics, Rockville, MD, USA). The examined brain regions are the CC, caudate-putamen (CPU), FC, and the CA3 of the hippocampus. Three sections were chosen for each brain region. Three images were recorded from each section under the same conditions. The data of MBP-like immunoreactivity were expressed as integrated optical density (IOD), which equals to area × average optical density. The number of GST-π and IBA-1 positive cells was counted and expressed as N/mm^2^.

### Enzyme-Linked Immunosorbent Assay

The protein levels of the cytokines IL-1β, IL-6, and IL-10 in the brain regions of CPU, FC, and hippocampus were measured using the ELISA kits (Boster, Wuhan, China) by following the procedures recommended by the manufacturer. Briefly, the brain tissue samples were homogenized in PBS (0.01 M, pH = 7.4) at 10% (w/v), followed by centrifugation at 3,000 rpm for 15 min at 4°C. Then, the supernatant was collected and stored at −80°C until use. On the analysis day, the samples were thawed and diluted with the dilute solution (1 volume sample + 9 volumes of dilute solution). Subsequently, the standard and sample solutions were added into the ELISA plates in duplicate (2 wells/solution, 100 μl/well) and incubated for 90 min at 37°C. Then, the liquid was thrown away, and 100 μl of biotin-labeled anti-mouse IL-1β, IL-6, or IL-10 working solution was added into the wells and incubated for 60 min at 37°C. After rinsed with PBS three times, 100 μl ABC (avidin-biotin-peroxidase complex) working solution was added into each well and incubated for 30 min at 37°C. Again, the liquid in wells was thrown away, and the wells were rinsed with PBS three times, followed by the addition of 90 μl TMB (3,3′,5,5′-tetramethylbenzidine) working solution. After incubation for 20–25 min at 37°C in the dark, the reaction was stopped by the addition of 100 μl TMB buffer. The plates were read by the microplate reader (Bio-Rad Laboratories Inc., Hercules, CA, USA) at 450 nm. The protein levels of the cytokines in the tissue samples were calculated by referring to the standard curve and expressed as pg/mg protein.

### Statistical Analyses

Excel 2010 and SPSS 20.0 (IBM Corp., Armonk, NY, USA) was used for data analysis. Data were expressed as means ± SD. The Shapiro–Wilk test was used for the normality test of data distribution. The interaction time of the tested mouse in each group under the two conditions of E and C was compared by paired *t*-test. For the data from the other analyses, one-way ANOVA was conducted before the Tukey-post test for comparison of two groups. The significance threshold was set at 0.05.

## Results

### Deep rTMS Alleviates the CPZ-Induced Anxiety-Like Behavior and Deficits in Social Recognition and Spatial Working Memory

The open-field test was performed with each one of the mice in this study to measure the locomotor activity, exploratory behavior, and anxiety level of the mice. Table 1 showed the results of the test. For TD, one-way ANOVA showed a significant effect of the treatments (*F*_(3,27)_ = 6.880, *p* = 0.002). The *post hoc* comparisons, however, did not find any significant difference between any two groups. Regarding CD, one-way ANOVA showed no significant effect of the treatments (*F*_(3,27)_ = 2.758, *p* = 0.064). As for CD/TD, one-way ANOVA showed a significant effect of the treatments (*F*_(3,27)_ = 3.103, *p* = 0.046). The results of *post hoc* comparisons indicated that CPZ-feeding decreased the ratio of CD/TD compared to the CNT group (*p* < 0.05); and the difference between groups CPZ + Sham and CPZ + rTMS was also significant (*p* < 0.05), suggesting a protective effect of deep rTMS on the CPZ-induced anxiety-like behavior in mice.

**Table 1:** Repetitive transcranial magnetic stimulation (rTMS) alleviates cuprizone (CPZ)-induced anxiety behavior in C57BL/6 mice.

Groups	TD (mean ± SD; cm)	CD (mean ± SD; cm)	CD/TD (mean ± SD; %)
CNT	1,723.46 ± 189.15	382.08 ± 59.99	22.17 ± 1.28
CPZ	2,137.64 ± 185.01	365.91 ± 27.60	17.12 ± 1.10*
CPZ + Sham	1,859.21 ± 94.99	332.48 ± 26.14	17.91 ± 1.39*
CPZ + rTMS	1,684.11 ± 103.28	398.25 ± 32.79	23.63 ± 1.20^#^

Note: *TD*, the total distance traveled on the whole arena of the open-field during a 5-min test period. *CD*, the distance traveled at the central part of the open-field arena during the test period. *p < 0.05, compared to CNT group. ^#^p < 0.05, compared to CPZ group.

**Figure 1:**
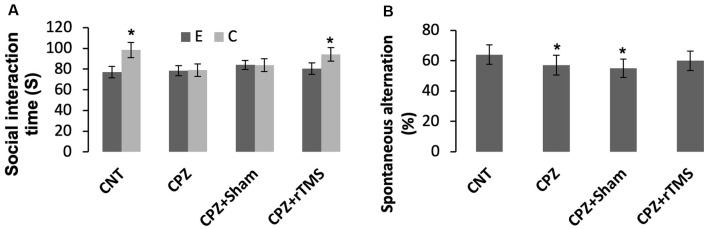
Repetitive transcranial magnetic stimulation (rTMS) improves the behavioral performance of cuprizone (CPZ)-fed mice. **(A)** rTMS improves the performance of CPZ-fed mice in the social interaction test. **(B)** rTMS improves the working memory of CPZ-fed mice. Data are expressed as mean ± SD. *N* = 14/group. *p < 0.05, compared to E or compared to CNT.

As for social recognition, mice in the groups CNT and CPZ + rTMS were able to tell an empty mesh cage from the same cage with an unfamiliar conspecific inside as evidenced by much more time spent by the mice under the latter condition relative to that spent under the former condition (*p* < 0.05). However, mice in groups of CPZ and CPZ + Sham were unable to tell the difference between the two different conditions (Figure 1A).

The Y-maze test was employed to measure spatial working memory and the exploratory behavior of mice. One-way ANOVA showed no significant effect of the treatments on arm visiting number (*F*_(3,55)_ = 1.502, *p* = 0.225), and no effect on number of correct visiting (*F*_(3,55)_ = 1.502, *p* = 0.225). As for the spontaneous alternation, one-way ANOVA showed a marginal effect of the treatments (*F*_(3,55)_ = 1.914, *p* = 0.106). *Post hoc* comparisons showed significant differences between CNT and CPZ groups, and between CNT and CPZ + Sham groups, but no difference between CNT and CPZ + rTMS groups (Figure 1B). The results suggest that rTMS blocked CPZ-induced decrease in spontaneous alternation.

### Deep rTMS Attenuates the CPZ-Induced Myelin Breakdown and OL Loss in Mice

**Figure 2:**
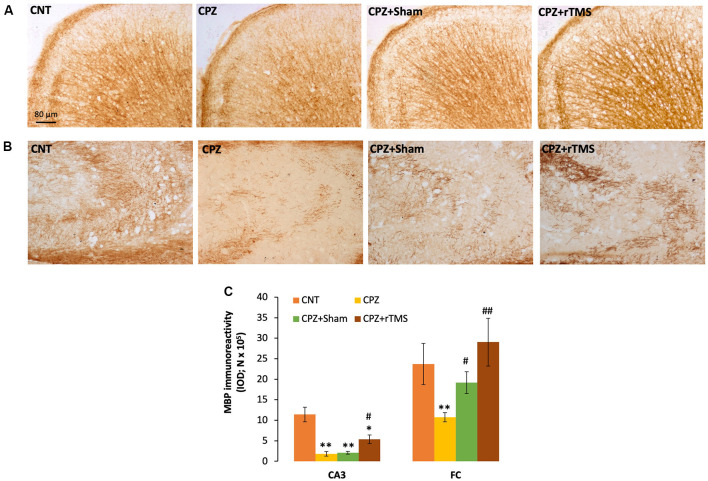
rTMS alleviates the myelin breakdown in CA3 and frontal cortex (FC) of CPZ-fed mice. **(A,B)** Representative images show myelin basic protein (MBP) immunoreactivity in FC and CA3 of the hippocampus of mice from the four groups of CNT, CPZ, CPZ + Sham, and CPZ + rTMS, respectively. **(C)** The bar chart shows the statistical analytic results of quantified MBP immunoreactivity in the two brain regions of mice. Data are expressed as mean ± SD. *N* = 7/group. *p < 0.05, **p < 0.01, compared to CNT. ^#^*p* < 0.05, ^##^*p* < 0.01, compared to CPZ.

We measured and compared the MBP immunoreactivity in the FC and hippocampus of all mice in this study. It seemed that CPZ-feeding led to myelin breakdown in both FC and CA3 of the hippocampus, and this disruption was also seen in the mice of the CPZ + Sham group (Figures 2A,B). One-way ANOVA on the quantitative data indicated a significant effect of the treatments on MBP immunoreactivity in FC (*F*_(3,23)_ = 3.546, *p* = 0.033). *Post hoc* comparisons showed a significant difference between CNT and CPZ groups, but no difference between CNT and CPZ + Sham groups and between CNT and CPZ + rTMS groups. The differences between CPZ and CPZ + Sham groups and between CPZ and CPZ + rTMS groups are also significant (Figure 2C). As for the IOD of MBP immunoreactivity in CA3, one-way ANOVA indicated a significant effect of the treatments (*F*_(3,23)_ = 16.925, *p* < 0.0001). *Post hoc* comparisons showed significant differences between CNT and each of the other three groups. Also, the differences between the CPZ + rTMS group and CPZ or CPZ + Sham group are significant (Figure 2C).

**Figure 3:**
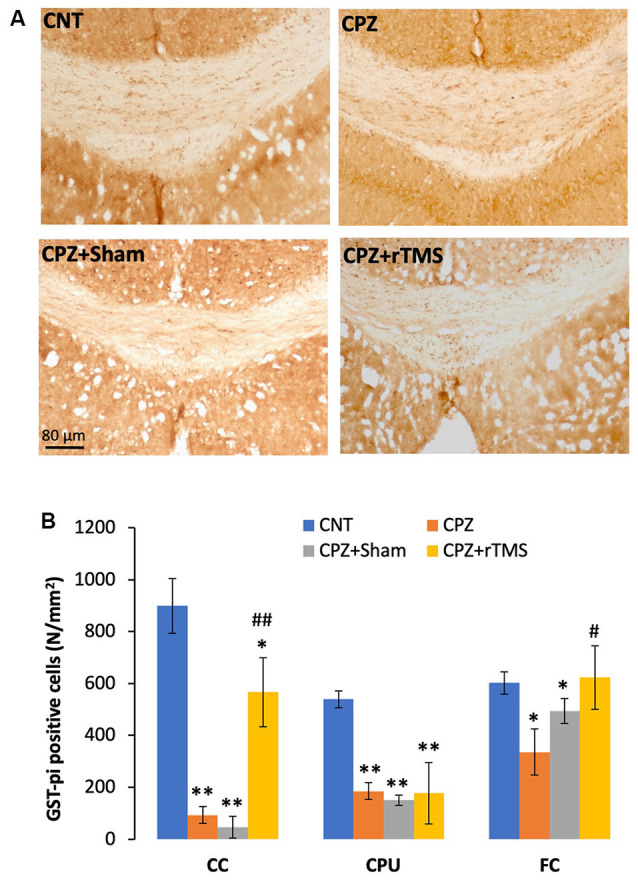
rTMS alleviates OLs loss in corpus callosum (CC), caudate-putamen (CPU), and FC of CPZ-fed mice. **(A)** Representative images show glutathione-S-transferase (GST-π) positive cells in CC of mice from the four groups. **(B)** The bar chart shows the statistical analytic results of GST-π positive cell number in the three brain regions of the mice. Data are expressed as mean ± SD. *N* = 7/group. *p < 0.05, **p < 0.01, compared to CNT. ^#^*p* < 0.05, ^##^*p* < 0.01, compared to CPZ.

In addition to demyelination and myelin breakdown, CPZ-feeding also led to OL loss in CC, CPU, and FC of the mouse brain. To check if deep rTMS applied in this study can reduce the CPZ-induced OL loss, we labeled mature OLs using the specific antibody to GST-π, a cytosolic isoenzyme used as a marker for mature OLs in the mammalian brain. Figure 3A showed many more GST-π positive cells in CC of the mice in the CNT group, whereas the cells were rarely seen in the same region of mice in the other three groups. One-way ANOVA against the quantitative data indicated a significant effect of the treatments on number of GST-π positive cells in the examined brain regions of CC (*F*_(3,27)_ = 6.155, *p* = 0.003), CPU (*F*_(3,27)_ = 6.531, *p* = 0.002), and FC (*F*_(3,27)_ = 4.929, *p* = 0.008). *Post hoc* comparisons indicated: (1) GST-π cells in CC and CPU significantly decreased in all CPZ-feeding mice (CPZ, CPZ + Sham, and CPZ + rTMS groups), but the number in FC of CPZ + rTMS group was comparable to that of CNT group; (2) rTMS significantly alleviated the CPZ-induced decrease in GST-π cells in CC and FC (CPZ + rTMS vs. CPZ), but not in CPU where the number of GST-π cells in CPZ + rTMS was comparable to those in CPZ and CPZ + Sham groups (Figure 3B).

### Deep rTMS Blocks Microglia Activation and Expression Changes in Inflammatory Cytokines in CPZ-Fed Mice

Previous studies have shown microglia increase and activation along with OL loss in brains of CPZ-exposed mice (Zhang J. et al., 2018; Luo et al., 2020). The microglial activation may exacerbate OLs death and demyelination *via* releasing pro-inflammation cytokines such as IL-1β, IL-6, and TNF-α (Aryanpour et al., 2017; Zhang J. et al., 2018). Therefore, it is plausible to hypothesize that deep rTMS should reduce the CPZ-induced microglia activation and OL loss if it works. We labeled microglia using the antibody to IBA-1, a small protein specifically and constitutively expressed in all microglia (Imai et al., 1996; Ahmed et al., 2007).

**Figure 4:**
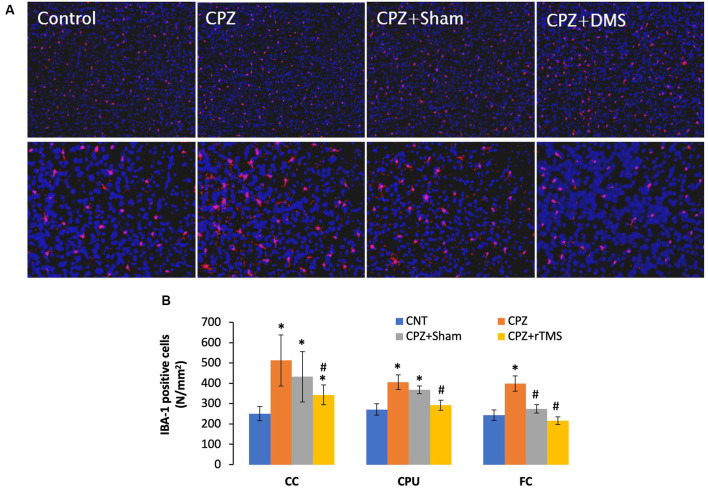
rTMS alleviates microglia increase in CC, CPU, and FC of CPZ-fed mice. **(A)** Representative images show IBA-1 positive cells in FC of mice from the four groups. **(B)** The bar chart shows the statistical analytic results of IBA-1 positive cell numbers in the three brain regions of the mice. Data are expressed as mean ± SD. *N* = 7/group. *p < 0.05, compared to CNT. ^#^*p* < 0.05, compared to CPZ.

Mice in the CPZ group showed many more IBA-1 positive cells in all three brain regions examined, but it was hard to tell differences among the other three groups based on qualitative observation only (Figure 4A). As such, we counted the number of IBA-1 positive cells and did comparisons on this parameter across all groups. One-way ANOVA results indicated a significant effect of the treatments on number of IBA-1 positive cells in the examined brain regions of CC (*F*_(3,27)_ = 8.311, *p* = 0.001), CPU (*F*_(3,27)_ = 7.807, *p* = 0.001), and FC (*F*_(3,27)_ = 3.112, *p* = 0.045). *Post hoc* comparisons indicated: (1) CPZ group had many more IBA-1 positive cells (CPZ vs. CNT) in all three regions examined; (2) rTMS significantly alleviated the CPZ-induced increases in IBA-1 positive cells in all the three regions (CPZ + rTMS vs. CPZ); and (3) FC is the only region showing comparable numbers of IBA-1 positive cell between CPZ + Sham and CNT group (Figure 4B).

**Figure 5:**
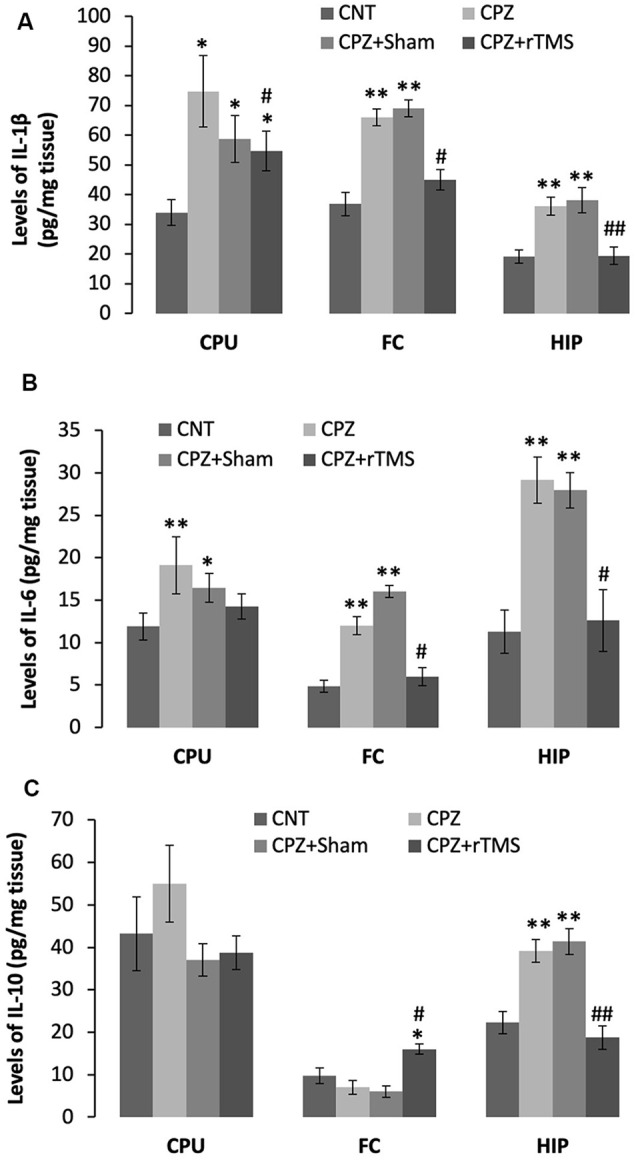
rTMS alleviates the CPZ-induced changes in expression levels of inflammatory cytokines in CPU, FC, and HIP of mice. **(A)** The bar chart shows the statistical analytic results of IL-1β levels in the three brain regions of the mice. **(B)** The bar chart shows the statistical analytic results of IL-6 levels in the three brain regions of the mice. **(C)** The bar chart shows the statistical analytic results of IL-10 levels in the three brain regions of the mice. Data are expressed as mean ± SD. *N* = 7/group. *p < 0.05, **p < 0.01, compared to CNT. ^#^*p* < 0.05, ^##^*p* < 0.01, compared to CPZ.

Furthermore, we measured some of the inflammatory cytokines in the brain regions of CPU, FC, and hippocampus of mice. The cytokines assessed in this study included pro-inflammatory ones of IL-1β and IL-6, plus IL-10, an anti-inflammatory one. One-way ANOVA results indicated a significant effect of the treatments on IL-1β levels in the examined brain regions of CPU (*F*_(3,27)_ = 4.374, *p* = 0.014), FC (*F*_(3,27)_ = 4.745, *p* = 0.01), and Hippocampus (*F*_(3,27)_ = 10.691, *p* < 0.0001). *Post hoc* comparisons indicated: (1) mice in CPZ and CPZ + Sham groups showed significantly higher IL-1β levels in CPU, FC, and hippocampus compared to CNT group; (2) no differences were found between CNT and CPZ + rTMS groups in this parameter in FC and hippocampus (Figure 5A).

As for IL-6 levels, one-way ANOVA results indicated a marginal effect of the treatments on this parameter in CPU (*F*_(3,27)_ = 2.305, *p* = 0.106), significant effects in FC (*F*_(3,27)_ = 3.833, *p* = 0.025) and Hippocampus (*F*_(3,27)_ = 8.908, *p* < 0.0001). *Post hoc* comparisons indicated: (1) mice in CPZ and CPZ + Sham groups showed significantly higher IL-6 levels in all the three brain regions compared to CNT group; and (2) no differences were found between CNT and CPZ + rTMS groups in this parameter (Figure 5B).

As for IL-10, one-way ANOVA results indicated no significant effect of the treatments on this parameter in CPU (*F*_(3,27)_ = 0.820, *p* = 0.496) and FC (*F*_(3,27)_ = 0.054, *p* = 0.983), but a significant effect in the Hippocampus (*F*_(3,27)_ = 11.342, *p* < 0.0001). *Post hoc* comparisons indicated: (1) all four groups showed comparable IL-10 levels in CPU; (2) the CPZ + rTMS group showed significantly higher IL-10 level than the other three groups; and (3) IL-10 level in the hippocampus was significantly higher in CPZ and CPZ + Sham groups compared to CNT group, but the CPZ + rTMS group was comparable to CNT group in this parameter (Figure 5C).

## Discussion

Following a recent study (Zhang J. et al., 2018), consuming a CPZ-containing diet for 5 weeks increased the anxiety level of mice in this study as evidenced by a significantly lower ratio of CD/TD in the CPZ group compared to the CNT group. This anxiety-like behavior was also seen in the CPZ + Sham group. However, rTMS treatment completely blocked the CPZ-induced anxiety in mice as the ratio of CD/TD in the CPZ + rTMS group was comparable to that in the CNT group, suggesting a protective effect of deep rTMS on the CPZ-induced anxiety in mice.

We noticed that the CPZ-containing diet did not change the performance of mice in the open-field test in some of the previous studies (Xu et al., 2009; Zhen et al., 2017). We compared the procedure details of the open-field tests performed in the present and previous studies and found that the open-field arena in previous studies was bigger (50 × 50 cm, or 53 × 53 cm) than that in the present study (25 × 25 cm). This difference may partly account for the inconsistency of the results from the present study and previous studies. In support of this speculation, the other two previous studies reported a decreased time and travel distance of CPZ-exposed mice in the central part of an open-field at dimensions of 25 × 25 cm (Zhang J. et al., 2018; Zhang L. et al., 2018). Therefore, the size of an open-field arena is an important factor influencing the performance of mice in this test.

Consuming a CPZ-containing diet for 5 weeks impaired the social recognition and spatial working memory of mice, which are consistent with the findings in previous studies (Xiao et al., 2008; Xu et al., 2009, 2010; Sun et al., 2018; Zhang J. et al., 2018). Moreover, the present study showed that rTMS completely blocked the CPZ-induced deficits in spatial memory and social recognition of mice, but the sham stimulation did not show this protective effect. In line with these results, a recent study reported that administration of high-frequency (gamma Hz) TBS for 6 weeks significantly alleviated schizophrenia-like behaviors in CPZ-fed mice, including improved nesting, social interaction, and sensorimotor gating, while low-frequency TBS only improved sensorimotor gating (Sun et al., 2018). Taken together, the data from the present and previous studies provided experimental evidence that deep rTMS is effective in preventing CPZ-induced behavioral abnormalities relevant to some of the symptoms seen in patients with schizophrenia.

Along with the behavioral improvement in CPZ-exposed mice, rTMS effectively alleviated CPZ-induced myelin breakdown and OL loss in CC, CPU, and hippocampus of mice in this study. These results are in line with a recent study in which both high-frequency and low-frequency TBS repaired the myelin sheath in CPZ-exposed mice (Sun et al., 2018). Relevantly, iGBS effectively enhanced neurogenesis in the dentate gyrus of mice while improved performance of the mice in spatial learning and memory (Zhang et al., 2014). Together, the results of the present study and previous studies suggest that deep rTMS affects a variety of target cells/cell structures in the brain thus improving higher functions of the brain including cognitive function and emotional function. Therefore, the application of deep rTMS to animal studies has specific relevance to the beneficial effects of this new modality in human patients with neuropsychiatric diseases and may help to reveal the underlying mechanisms for the therapeutic effects of rTMS.

Of the four brain regions examined in this study, we noticed that the CPZ-induced myelin breakdown and OL loss in FC were completely blocked in the CPZ + rTMS group whereas the blockade was incomplete in CPU and hippocampus. This region-specific effect of deep rTMS may be related to the anatomical position of these brain regions, of which CPU and hippocampus are underneath FC. Another possible mechanism may be attributed to a more efficient self-repairing capacity of FC relative to the CPU and hippocampus. This superior self-repairing capacity is associated with the up-regulated levels of IL-10, an anti-inflammatory cytokine, in this region (discussion later).

Previous studies (Zhang et al., 2008; Zhang J. et al., 2018; Zhang et al., 2019; Shao et al., 2015; Zhen et al., 2017; Roboon et al., 2019) have shown that demyelination and OL loss in CPZ-exposed mice are accompanied with astrocyte and microglia activation. The microglial activation appeared even in the absence of demyelination (Roboon et al., 2019) or in the non-myelinolysis regions of the brain (Zhang et al., 2019), suggesting that microglial activation was not initiated by demyelination in the CPZ-exposed mice of these previous studies. On the other hand, activated microglia and inflammatory cytokines released from these cells may induce damage to neighboring neurons and glia, of which OLs are particularly susceptible (Peferoen et al., 2014). For example, intracerebral injection of lipopolysaccharide in young rats invokes activation of microglia and production of pro-inflammatory cytokines that ultimately leads to hypomyelination (Pang et al., 2003; Chew et al., 2013). Moreover, activated microglia arrest oligodendrocyte progenitor cell (OPC) proliferation and induce OPC death as seen in previous *in vitro* studies (Sherwin and Fern, 2005; Li et al., 2008). Considering all these previous findings, it is plausible to claim that deep rTMS alleviates myelin breakdown and OL loss *via* inhibiting microglia activation in the CPZ-exposed mice in the present study. In line with this interpretation, the alleviation of myelin breakdown and OL loss was parallel to the inhibition of microglia activation in the present study. Levels of the pro-inflammatory cytokines IL-1β and IL-6 increased in CPU, CTX, and hippocampus of all mice in CPZ and CPZ + Sham groups relative to the CNT group while these CPZ-exposed mice showed obvious myelin breakdown and OL loss. But, levels of these cytokines in the CPZ + rTMS group were comparable to those in the CNT group, in parallel to alleviated myelin breakdown and OL loss in the examined regions. Relevant to the aforementioned findings in this study, a most recent animal study reported an anti-inflammation action of rTMS as evidenced by reduced levels of TNF-α, iNOS, IL-1β, and IL-6 in the hippocampus, and the decreases were associated with the antidepressant and anxiolytic-like effects in rats (Tian et al., 2020).

IL-10 is a pleiotropic cytokine showing a broad spectrum of anti-inflammatory properties (Moore et al., 2001). Previous human studies have associated lower IL-10 levels with MS severity and with the progressive stage of the disease (van Boxel-Dezaire et al., 1999; Petereit et al., 2003; Soldan et al., 2004). In a recent study, IL-10 suppressed the response of cultured microglia to recombinant granulocyte-macrophage colony-stimulating factor (Mayo et al., 2016). In the present study, consuming CPZ- containing diet for 5 weeks increased IL-10 level in the hippocampus, but not in CPU and FC, compared to the CNT group. Deep rTMS increased IL-10 level in FC, but not in CPU and hippocampus. These results are in line with a recent study, in which CPZ-treated mice showed a higher level of IL-10 in the hippocampus, but lower in CTX. The same study also showed that adenosine (10 mg/kg) increased IL-10 level in CTX but had no effect on IL-10 level in the hippocampus while promoted the remyelination process in the two brain regions of CPZ-treated mice (Zhang J. et al., 2018). Taken together, we may conclude that alleviations of CPZ-induced myelin breakdown and OL loss in FC by deep rTMS (in the present study) and adenosine (in a previous study) were associated with the up-regulation of IL-10 in this region while CPU and hippocampus did not show this up-regulation in response to deep rTMS and adenosine. Further studies are needed to address why these brain regions are different in terms of IL-10 levels in response to CPZ-exposure and rTMS treatment.

We are aware of the limitations of this study. First, it would be best to include two more animal groups of Control-sham and Control-rTMS to examine the possible effects of rTMS on normal healthy controls. Second, depressive-like behaviors could be tested if another batch of animals were included. Third, this study could not determine a causal relationship between microglial activation and levels of IL-1β, IL-6, and IL-10 in the examined regions of the mouse brain. And the role of IL-10 is undetermined. Fourth, there was no significant difference between CPZ + Sham and CPZ + rTMS groups regarding most of the examined indices in CPU, including numbers of GST-π and IBA-1 positive cells, as well as levels of IL-1β, IL-6, and IL-10. This is not surprising given that remyelination occurs spontaneously in demyelinated sites of CPZ-exposed mice. However, this phenomenon cannot be regarded as the argument against the conclusion that the administration of deep rTMS is of help in reducing the CPZ-induced neuropathological changes and/or promoting the recovery process of the subjects from the brain damage thus protecting the brain functions.

In conclusion, consuming a CPZ-containing diet for 5 weeks induced social interaction and spatial memory deficits and increased anxiety level in mice, along with myelin breakdown and OL loss in CC, CPU, FC, and hippocampus of the mouse brain. Deep rTMS for 4 weeks during the CPZ-exposure period effectively blocked the behavioral changes, myelin breakdown, and OL loss in CPZ-fed mice while inhibited microglia activation in the lesion sites and regulated the expression of inflammatory cytokines of IL-1β, IL-6, and IL-10 in the same brain regions. Relative to the other brain regions, FC is one that was protected most effectively by deep rTMS in terms of neuropathological parameters. This region-specific difference may be related to the anatomical position of FC being over CPU and hippocampus and associated with the upregulated expression of IL-10 in this region. These data added experimental evidence for the beneficial effects of deep rTMS in treating behavioral abnormalities and improving the associated neuropathologic damage to the brain. Moreover, CPZ-exposed mice are a suitable animal model for exploring the neurobiological mechanisms underlying the beneficial effects of this non-invasive physical therapy in treating some of the neuropsychiatric disorders. In such contexts, this study is of particular relevance to the treatment of neuropsychiatric disorders, for which many extant medications are unsatisfactory and new therapeutic modalities are needed.

## Data Availability Statement

The raw data supporting the conclusions of this article will be made available by the authors, without undue reservation.

## Ethics Statement

The animal study was reviewed and approved by Shantou University Medical College.

## Author Contributions

QH, LY, and HX conceived the study idea and designed the experiments. LY, YS, and FG carried out experiments and analyzed the data. YZ and HZ instructed the experiments and data analysis. LY and HX interpreted the results and wrote the manuscript. QH and HX revised the manuscript. All authors contributed to the article and approved the submitted version.

## Conflict of Interest

The authors declare that the research was conducted in the absence of any commercial or financial relationships that could be construed as a potential conflict of interest.
